# Supplementary material for: Survival in Colon, Rectal and Small Intestinal Cancers in the Nordic Countries through a Half Century
Source: Cancers (Basel). 2023 Feb 3;15(3):991. doi: 10.3390/cancers15030991 (PMC9913304; doi:10.3390/cancers15030991)
Supplement: Supplementary file 1 [file cancers-15-00991-s001.zip › 22intestinalCAsupplementery InformationCANCERSs.pdf]

SURVIVAL IN COLON, RECTAL AND SMALL INTESTINAL CANCERS IN THE NORDIC COUNTRIES THROUGH A HALF CENTURY

Filip Tichanek, Asta Försti, Akseli Hemminki, Kari Hemminki

Supplementary information

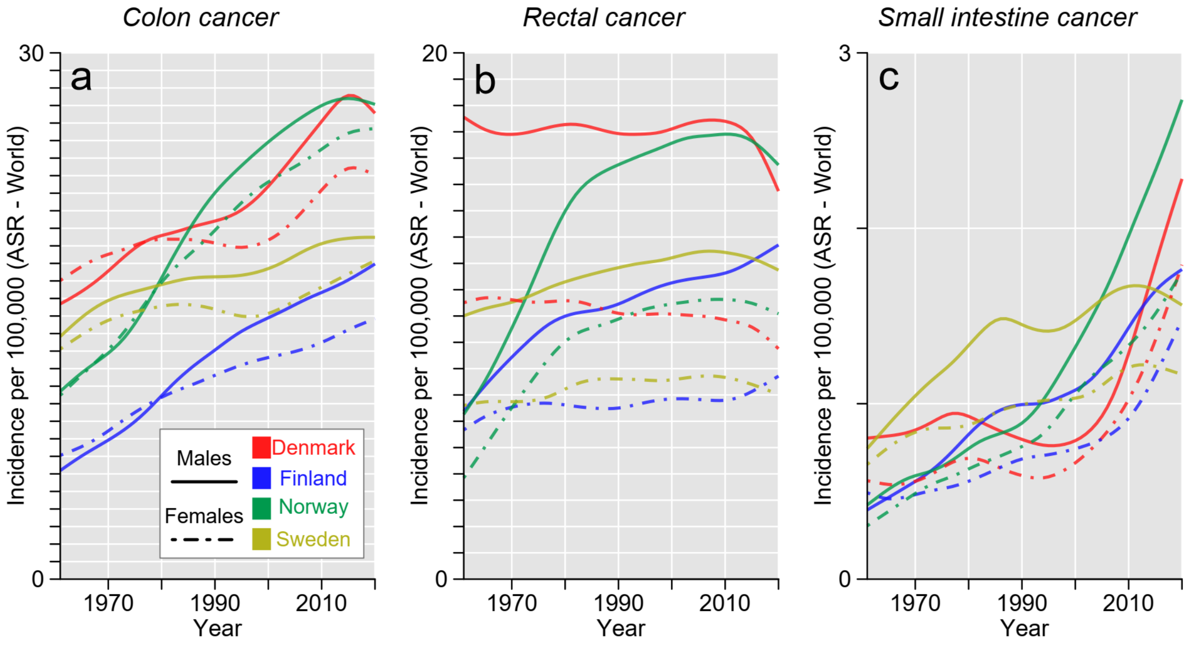

Supplementary Fig. 1. Incidence of colon (a), rectal (b) and small intestine (c) cancers from 1961 to 2020 in Denmark, Finland, Norway and Sweden, separately for males and females. Lines were smoothed via cubic smoothing spline.

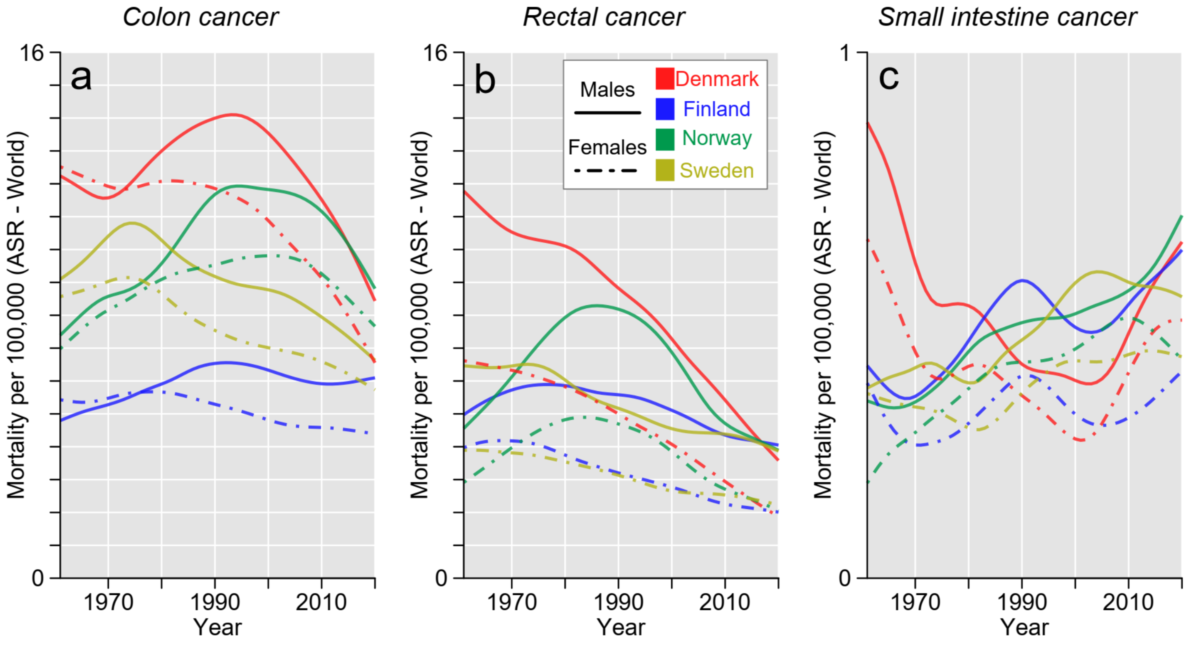

Supplementary Fig. 2. Mortality in colon (a), rectal (b) and small intestine (c) cancer patients from 1961 to 2020 in Denmark, Finland, Norway and Sweden, separately for males and females. Lines were smoothed via cubic smoothing spline.

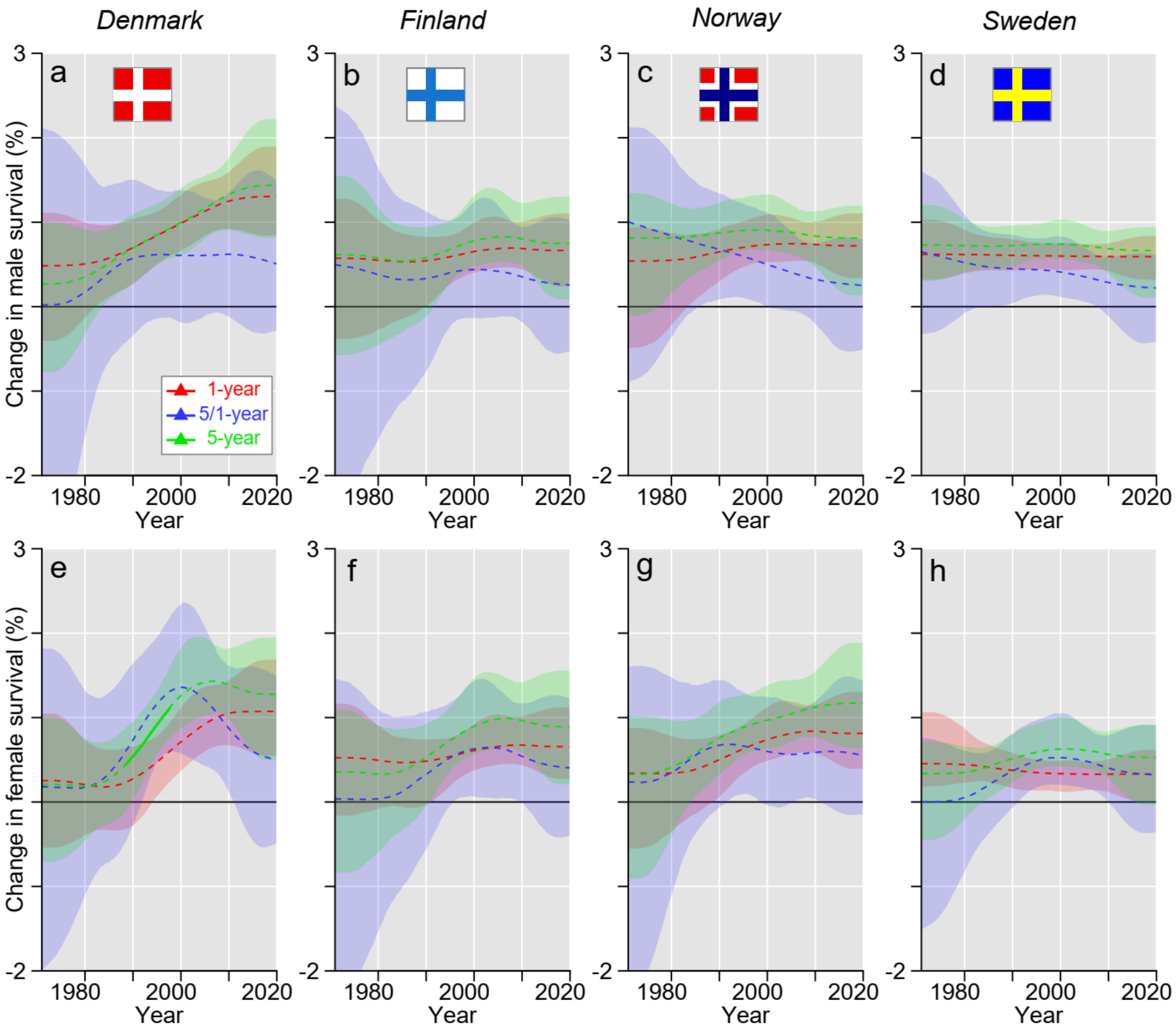

Supplementary Fig 3. The magnitude of change (per a year) in 1-year, 5-years and 5/1-year (4-years conditional) relative survival in small intestine cancer patients, separately for males (a-d) and females (e-h). Solid lines indicate at least 95% plausibility for the trend. All curves are color coded (see the insert).

Supplementary Table 1. 1-year relative survival [95% confidence interval] in colon, rectal and small intestine cancers from 1971 to 2020. *Significant increase between the marked and the next period.

**Male cancers**					**Female cancers**			
**Colon**	Denmark	Finland	Norway	Sweden	Denmark	Finland	Norway	Sweden
1971-1975	57.4 [55.5-59.4]*	54.3 [50.8-57.9]*	57.1 [54.8-59.5]*	58.3 [56.9-59.8]*	60.3 [58.6-62.1]	57.5 [55.1-60.1]*	61.7 [59.7-63.8]*	62.8 [61.5-64.1]*
1976-1980	60.4 [58.7-62.1]	60.1 [57.2-63.2]*	65.8 [63.8-67.8]*	66.2 [64.8-67.6]*	61.4 [59.9-62.9]*	62.3 [60.1-64.5]*	65.6 [63.9-67.4]*	68.7 [67.5-70.0]*
1981-1985	61.7 [60.0-63.4]*	68.4 [66.0-70.9]	70.9 [69.2-72.7]	71.6 [70.3-72.9]*	64.3 [62.9-65.8]*	67.6 [65.7-69.6]*	71.9 [70.4-73.5]	73.6 [72.5-74.8]*
1986-1990	66.2 [64.7-67.9]	68.3 [66.1-70.6]*	69.9 [68.2-71.6]*	73.1 [71.8-74.3]*	67.8 [66.4-69.2]*	71.8 [70.0-73.6]*	72.4 [70.9-73.9]*	76.0 [74.9-77.1]*
1991-1995	67.0 [65.5-68.6]*	74.5 [72.6-76.4]*	72.3 [70.8-73.9]*	75.8 [74.6-76.9]*	70.0 [68.6-71.4]*	73.9 [72.3-75.6]*	75.6 [74.2-76.9]*	78.3 [77.3-79.4]*
1996-2000	69.8 [68.4-71.3]*	77.1 [75.3-78.8]*	76.6 [75.2-78.0]	78.9 [77.8-80.0]*	72.2 [70.9-73.6]*	79.1 [77.6-80.6]*	79.5 [78.3-80.7]	80.4 [79.4-81.4]*
2001-2005	72.3 [71.0-73.7]*	78.9 [77.4-80.4]*	77.7 [76.4-79.0]*	81.6 [80.6-82.6]*	75.2 [74.0-76.4]*	81.8 [80.5-83.2]	79.7 [78.6-80.9]*	82.1 [81.2-83.1]*
2006-2010	77.0 [75.8-78.1]*	81.4 [80.1-82.7]*	80.1 [79.0-81.3]*	83.7 [82.8-84.6]	78.0 [76.9-79.1]*	83.2 [82.0-84.4]	81.8 [80.7-82.9]*	84.3 [83.5-85.2]
2011-2015	83.7 [82.8-84.6]*	83.0 [81.8-84.2]	83.1 [82.1-84.1]*	84.4 [83.6-85.3]*	84.3 [83.4-85.2]*	83.8 [82.7-85.0]*	85.1 [84.2-86.0]*	84.7 [83.9-85.5]*
2016-2020	87.3 [86.5-88.1]	83.0 [81.9-84.1]	86.9 [86.0-87.8]	85.9 [85.2-86.7]	86.6 [85.8-87.4]	85.8 [84.8-86.8]	86.5 [85.6-87.3]	85.8 [85.1-86.6]
**Rectum**								
1971-1975	65.5 [63.6-67.5]	65.2 [62.0-68.6]*	66.6 [64.0-69.3]*	68.7 [67.0-70.4]*	67.9 [65.8-70.1]	68.3 [65.5-71.2]	67.3 [64.6-70.1]*	68.8 [67.0-70.7]*
1976-1980	65.9 [64.1-67.8]*	72.6 [70.0-75.4]*	70.8 [68.6-72.9]*	71.4 [69.8-73.0]*	68.4 [66.5-70.4]*	72.0 [69.6-74.5]	74.0 [71.9-76.2]*	75.3 [73.6-77.1]*
1981-1985	69.2 [67.4-71.0]*	75.9 [73.5-78.4]	73.9 [72.1-75.9]*	74.9 [73.4-76.5]*	71.2 [69.3-73.0]	73.5 [71.1-75.9]*	77.0 [75.0-79.0]	78.1 [76.7-79.7]*
1986-1990	71.5 [69.7-73.2]*	77.7 [75.4-80.1]	77.1 [75.3-78.9]*	77.4 [76.0-78.8]*	71.2 [69.4-73.1]*	76.5 [74.3-78.8]*	78.7 [76.8-80.7]*	80.1 [78.7-81.6]*
1991-1995	74.0 [72.4-75.6]	76.7 [74.6-78.8]*	79.8 [78.2-81.5]	80.1 [78.8-81.4]*	76.4 [74.6-78.2]	80.1 [78.1-82.1]*	82.0 [80.3-83.6]	82.1 [80.8-83.5]*
1996-2000	75.5 [73.9-77.1]*	80.8 [78.9-82.8]	81.0 [79.4-82.7]*	83.7 [82.5-84.8]	77.9 [76.2-79.5]*	82.8 [81.0-84.7]	83.5 [81.9-85.2]*	84.2 [82.9-85.5]*
2001-2005	80.1 [78.7-81.5]*	81.4 [79.7-83.3]*	83.3 [81.9-84.7]*	83.9 [82.8-85.0]*	79.9 [78.3-81.6]*	82.9 [81.2-84.7]*	85.5 [84.1-87.1]*	86.2 [85.1-87.4]*
2006-2010	83.7 [82.5-85.0]*	83.7 [82.2-85.3]	86.6 [85.4-87.9]*	86.2 [85.2-87.2]*	83.3 [81.9-84.8]*	86.5 [85.0-88.1]	87.6 [86.3-89.0]*	86.9 [85.8-88.1]*
2011-2015	87.5 [86.5-88.6]*	85.1 [83.8-86.5]*	89.0 [87.9-90.2]	87.9 [87.0-88.8]*	87.8 [86.5-89.0]*	86.5 [85.0-88.0]*	89.4 [88.1-90.6]*	89.0 [88.0-90.1]
2016-2020	89.6 [88.6-90.6]	87.3 [86.1-88.5]	89.5 [88.4-90.5]	88.9 [88.0-89.8]	90.6 [89.4-91.7]	88.6 [87.3-89.9]	90.9 [89.7-92.1]	89.2 [88.2-90.2]
**Small Intestine**								
1971-1975	49.1 [39.5-61.1]		62.8 [50.8-77.5]	52.4 [46.6-58.8]*	55.2 [45.7-66.7]	56.1 [43.8-71.8]	63.4 [53.6-74.8]	60.3 [54.6-66.6]*
1976-1980	37.6 [30.4-46.4]*	57.6 [45.8-72.4]	57.5 [47.7-69.4]	67.1 [61.0-73.7]	47.1 [39.1-56.7]*	68.4 [58.1-80.4]	61.8 [51.7-73.9]	70.8 [65.0-77.2]
1981-1985	52.3 [43.6-62.6]	65.6 [56.3-76.5]	64.6 [56.9-73.4]	63.0 [57.9-68.6]	63.6 [56.0-72.3]	69.6 [60.9-79.6]	57.3 [49.3-66.7]*	67.0 [61.7-72.7]
1986-1990	48.3 [40.4-57.9]	68.5 [60.2-77.9]	55.6 [46.8-66.0]	61.5 [56.5-66.9]*	52.1 [44.0-61.8]	68.4 [60.7-77.1]	69.9 [62.2-78.6]	71.5 [67.0-76.4]
1991-1995	52.8 [45.0-61.8]	56.2 [48.2-65.6]*	65.9 [58.2-74.5]	71.8 [67.5-76.3]	56.7 [48.4-66.4]	65.8 [58.1-74.5]	61.8 [54.4-70.2]	76.1 [72.0-80.5]
1996-2000	54.1 [46.4-63.2]	71.1 [63.9-79.0]	68.2 [61.5-75.5]*	71.4 [67.4-75.7]	52.1 [44.6-60.7]*	67.0 [59.4-75.6]*	66.9 [60.3-74.2]	73.1 [69.0-77.5]
2001-2005	60.7 [53.6-68.8]	69.9 [62.9-77.6]	76.9 [71.5-82.7]	75.6 [72.0-79.4]*	64.0 [56.6-72.3]	78.5 [72.5-85.1]	72.7 [67.1-78.9]	76.1 [72.1-80.2]
2006-2010	67.2 [60.8-74.3]*	75.8 [70.3-81.7]*	80.6 [76.0-85.4]	79.7 [76.6-83.0]	68.5 [62.4-75.3]	77.2 [71.7-83.1]*	78.8 [73.9-83.9]	77.7 [74.3-81.2]
2011-2015	75.1 [70.7-79.7]*	81.8 [77.6-86.3]	80.1 [76.2-84.3]*	79.3 [76.3-82.5]*	72.3 [67.0-77.9]*	84.0 [79.8-88.5]	82.8 [78.7-87.2]	80.4 [77.3-83.6]
2016-2020	81.4 [78.2-84.8]	82.6 [79.0-86.4]	86.7 [83.6-89.8]	84.9 [82.2-87.7]	79.3 [75.6-83.2]	86.4 [83.0-90.0]	86.3 [83.0-89.8]	80.9 [77.8-84.2]

Supplementary Table 2. 5-year relative survival [95% confidence interval] in colon, rectal and small intestine cancers from 1971 to 2020. *Significant increase between the marked and the next period.

**Male cancers**					**Female cancers**			
**Colon**	Denmark	Finland	Norway	Sweden	Denmark	Finland	Norway	Sweden
1971-1975	35.6 [33.5-37.9]	34.5 [30.9-38.6]*	39.7 [37.0-42.6]*	38.4 [36.8-40.2]*	38.3 [36.4-40.2]	35.9 [33.3-38.6]*	41.1 [38.8-43.5]*	42.4 [40.9-44.0]*
1976-1980	38.5 [36.5-40.6]	39.5 [36.2-43.1]*	43.4 [41.0-45.9]*	45.8 [44.1-47.6]*	39.2 [37.5-40.9]*	40.1 [37.8-42.5]*	44.8 [42.8-46.9]*	47.7 [46.2-49.3]*
1981-1985	37.7 [35.7-39.7]*	47.7 [44.7-51.0]	49.1 [46.9-51.5]	49.1 [47.4-50.8]*	42.3 [40.6-44.1]*	46.8 [44.5-49.2]*	49.2 [47.3-51.2]*	54.0 [52.5-55.4]
1986-1990	42.3 [40.3-44.3]*	49.1 [46.2-52.2]*	47.8 [45.7-50.1]*	51.5 [49.9-53.2]*	45.6 [43.9-47.3]*	53.9 [51.7-56.2]	52.5 [50.7-54.4]	54.8 [53.4-56.2]*
1991-1995	44.9 [43.0-46.9]*	54.9 [52.2-57.7]*	50.2 [48.3-52.3]*	55.5 [54.0-57.1]	48.2 [46.6-50.0]*	55.0 [52.9-57.2]*	54.3 [52.6-56.1]*	57.4 [56.0-58.9]*
1996-2000	48.4 [46.6-50.4]*	58.5 [56.0-61.0]	56.6 [54.7-58.6]	56.6 [55.1-58.2]*	50.8 [49.2-52.6]*	61.6 [59.6-63.6]*	59.1 [57.4-60.8]*	59.3 [57.9-60.8]*
2001-2005	53.1 [51.4-54.9]*	59.4 [57.3-61.7]*	58.1 [56.4-59.9]*	58.6 [57.2-60.0]*	57.4 [55.8-59.1]*	64.4 [62.6-66.3]	61.3 [59.7-62.9]*	63.5 [62.2-64.9]*
2006-2010	58.8 [57.3-60.4]*	63.8 [61.9-65.7]*	61.7 [60.1-63.4]*	64.5 [63.2-65.8]*	59.6 [58.1-61.1]*	64.6 [62.9-66.4]*	65.3 [63.8-66.9]*	67.0 [65.8-68.3]*
2011-2015	68.3 [66.8-69.7]*	66.1 [64.3-67.9]	65.1 [63.6-66.6]*	66.2 [65.0-67.5]*	69.3 [68.0-70.6]*	68.6 [67.0-70.3]	69.6 [68.3-71.0]*	68.5 [67.3-69.6]*
2016-2020	72.9 [71.6-74.2]	66.1 [64.5-67.8]	69.8 [68.4-71.2]	68.7 [67.5-69.8]	73.0 [71.7-74.2]	69.9 [68.3-71.5]	72.0 [70.7-73.3]	70.6 [69.5-71.7]
**Rectum**								
1971-1975	35.0 [32.8-37.3]	31.1 [27.7-34.9]*	33.9 [31.0-37.1]*	36.2 [34.2-38.3]*	37.2 [34.9-39.8]*	35.4 [32.3-38.7]*	38.7 [35.6-42.1]*	40.8 [38.7-43.1]*
1976-1980	35.8 [33.7-38.0]	39.1 [35.9-42.5]	39.8 [37.1-42.6]*	40.7 [38.8-42.8]*	40.4 [38.1-42.8]	41.4 [38.6-44.3]	46.0 [43.3-49.0]*	45.7 [43.6-48.0]*
1981-1985	36.6 [34.6-38.8]*	42.3 [39.2-45.8]*	44.4 [41.9-47.0]*	45.1 [43.1-47.1]*	41.0 [38.8-43.3]	43.8 [41.0-46.9]*	49.1 [46.5-51.8]*	49.8 [47.8-51.9]*
1986-1990	39.6 [37.5-41.8]*	47.9 [44.7-51.3]	47.4 [45.0-50.0]*	47.7 [45.8-49.6]*	42.2 [40.0-44.5]*	48.6 [45.7-51.7]*	52.9 [50.3-55.7]*	52.3 [50.3-54.4]*
1991-1995	43.8 [41.8-46.0]*	49.9 [47.0-52.9]*	51.6 [49.2-54.1]*	53.9 [52.0-55.8]*	47.8 [45.5-50.2]*	54.4 [51.6-57.3]*	57.9 [55.5-60.4]*	58.5 [56.6-60.5]*
1996-2000	47.6 [45.5-49.8]*	56.1 [53.3-59.1]*	57.4 [55.0-59.9]*	57.2 [55.4-59.0]	50.8 [48.5-53.2]*	58.5 [55.9-61.2]*	61.2 [58.8-63.6]*	61.5 [59.6-63.5]*
2001-2005	56.0 [54.0-58.1]*	59.4 [56.8-62.0]*	60.8 [58.7-63.0]*	58.7 [57.0-60.4]*	55.9 [53.6-58.2]*	62.7 [60.2-65.2]*	67.2 [64.9-69.5]	64.4 [62.6-66.2]
2006-2010	61.6 [59.7-63.6]*	64.3 [62.0-66.8]	67.3 [65.3-69.4]*	63.9 [62.3-65.6]*	64.2 [62.1-66.4]*	68.3 [66.0-70.8]*	68.2 [66.0-70.5]	65.5 [63.7-67.3]*
2011-2015	70.3 [68.5-72.1]	64.1 [61.9-66.3]*	70.0 [68.1-71.9]	67.8 [66.3-69.3]	71.9 [69.9-74.0]*	71.1 [68.8-73.4]*	70.2 [68.1-72.3]*	69.5 [67.8-71.2]
2016-2020	72.0 [70.3-73.7]	66.9 [64.9-69.1]	71.6 [69.8-73.4]	69.2 [67.7-70.7]	74.9 [73.0-76.9]	73.5 [71.4-75.7]	73.3 [71.2-75.3]	70.8 [69.2-72.6]
**Small Intestine**								
1971-1975	31.6 [21.9-45.6]		38.1 [24.6-58.9]	31.9 [25.9-39.2]*	24.9 [16.7-37.2]	37.0 [22.2-61.6]	41.6 [31.3-55.2]	41.6 [35.4-48.9]*
1976-1980	19.5 [13.4-28.5]	37.4 [26.2-53.3]	30.1 [20.0-45.5]	43.6 [35.7-53.4]	22.9 [16.4-32.2]*	46.0 [35.8-59.2]	33.6 [23.8-47.5]	50.3 [43.0-58.9]
1981-1985	27.3 [19.4-38.4]	47.5 [35.5-63.5]	37.9 [29.1-49.3]	43.6 [37.8-50.3]	32.5 [24.8-42.5]	47.7 [37.7-60.3]	37.0 [28.8-47.5]	43.1 [37.3-49.8]
1986-1990	27.9 [20.2-38.7]	52.9 [42.1-66.4]	39.3 [30.1-51.2]	41.1 [35.7-47.4]*	21.6 [15.0-31.2]	37.9 [29.6-48.5]	39.8 [31.1-50.8]	42.0 [36.8-48.0]*
1991-1995	35.4 [27.1-46.3]	32.8 [24.9-43.1]*	49.8 [40.7-60.8]	49.0 [43.4-55.4]	30.7 [22.8-41.4]	42.9 [34.5-53.4]	42.1 [34.2-51.7]	50.5 [45.3-56.3]
1996-2000	32.1 [24.6-42.0]	56.7 [47.0-68.3]	46.2 [38.0-56.1]*	51.6 [46.3-57.5]*	29.9 [22.9-38.9]*	46.8 [38.4-57.1]*	50.9 [43.4-59.8]	51.7 [46.7-57.2]
2001-2005	41.2 [33.5-50.7]	48.3 [40.2-57.9]*	56.3 [49.4-64.2]*	57.8 [53.0-63.0]	42.1 [34.1-51.9]*	58.1 [50.4-67.1]	55.6 [48.8-63.3]	56.4 [51.4-61.8]
2006-2010	46.3 [39.1-54.7]*	61.3 [54.1-69.5]	65.8 [59.6-72.6]	61.9 [57.6-66.5]	52.2 [45.3-60.3]	61.3 [54.5-69.1]	58.6 [52.4-65.5]	60.7 [56.4-65.4]
2011-2015	55.0 [49.3-61.3]*	63.3 [57.3-70.0]	63.5 [58.3-69.2]	62.9 [58.8-67.3]	54.7 [48.7-61.5]*	64.3 [58.3-70.9]	65.3 [59.4-71.6]*	61.7 [57.4-66.2]
2016-2020	62.3 [57.3-67.6]	65.1 [59.7-71.0]	67.9 [62.9-73.3]	66.0 [61.8-70.4]	62.1 [57.0-67.8]	70.3 [64.8-76.1]	72.9 [67.8-78.5]	64.1 [59.9-68.6]

Supplementary Table 3. 5/1-year (4-years conditional) survival in colon, rectal and small intestine cancers from 1971 to 2020.

Male cancers					Female cancers			
**Colon**	Denmark	Finland	Norway	Sweden	Denmark	Finland	Norway	Sweden
1971-1975	62.0	63.5	69.5	65.9	63.5	62.4	66.6	67.5
1976-1980	63.7	65.7	66.0	69.2	63.8	64.4	68.3	69.4
1981-1985	61.1	69.7	69.3	68.6	65.8	69.2	68.4	73.4
1986-1990	63.9	71.9	68.4	70.5	67.3	75.1	72.5	72.1
1991-1995	67.0	73.7	69.4	73.2	68.9	74.4	71.8	73.3
1996-2000	69.3	75.9	73.9	71.7	70.4	77.9	74.3	73.8
2001-2005	73.4	75.3	74.8	71.8	76.3	78.7	76.9	77.3
2006-2010	76.4	78.4	77.0	77.1	76.4	77.6	79.8	79.5
2011-2015	81.6	79.6	78.3	78.4	82.2	81.9	81.8	80.9
2016-2020	83.5	79.6	80.3	80.0	84.3	81.5	83.2	82.3
**Rectum**								
1971-1975	53.4	47.7	50.9	52.7	54.8	51.8	57.5	59.3
1976-1980	54.3	53.9	56.2	57.0	59.1	57.5	62.2	60.7
1981-1985	52.9	55.7	60.1	60.2	57.6	59.6	63.8	63.8
1986-1990	55.4	61.6	61.5	61.6	59.3	63.5	67.2	65.3
1991-1995	59.2	65.1	64.7	67.3	62.6	67.9	70.6	71.3
1996-2000	63.0	69.4	70.9	68.3	65.2	70.7	73.3	73.0
2001-2005	69.9	73.0	73.0	70.0	70.0	75.6	78.6	74.7
2006-2010	73.6	76.8	77.7	74.1	77.1	79.0	77.9	75.4
2011-2015	80.3	75.3	78.7	77.1	81.9	82.2	78.5	78.1
2016-2020	80.4	76.6	80.0	77.8	82.7	83.0	80.6	79.4
**Small Intestine**								
1971-1975	64.4		60.7	60.9	45.1	66.0	65.6	69.0
1976-1980	51.9	64.9	52.3	65.0	48.6	67.3	54.4	71.0
1981-1985	52.2	72.4	58.7	69.2	51.1	68.5	64.6	64.3
1986-1990	57.8	77.2	70.7	66.8	41.5	55.4	56.9	58.7
1991-1995	67.0	58.4	75.6	68.2	54.1	65.2	68.1	66.4
1996-2000	59.3	79.7	67.7	72.3	57.4	69.9	76.1	70.7
2001-2005	67.9	69.1	73.2	76.5	65.8	74.0	76.5	74.1
2006-2010	68.9	80.9	81.6	77.7	76.2	79.4	74.4	78.1
2011-2015	73.2	77.4	79.3	79.3	75.7	76.5	78.9	76.7
2016-2020	76.5	78.8	78.3	77.7	78.3	81.4	84.5	79.2
